# Increased temperatures and elevated CO_2_ levels reduce the sensitivity of *Conyza canadensis* and *Chenopodium album* to glyphosate

**DOI:** 10.1038/s41598-019-38729-x

**Published:** 2019-02-18

**Authors:** Maor Matzrafi, Caio Brunharo, Parsa Tehranchian, Bradley D. Hanson, Marie Jasieniuk

**Affiliations:** 1 Department of Plant Sciences, University of California-Davis, Davis, CA 95616 USA; 2 SynTech Research, P.O. Box 700, Sanger, CA 93657 USA

## Abstract

Herbicides are the most commonly used means of controlling weeds. Recently, there has been growing concern over the potential impacts of global climate change, specifically, increasing temperatures and elevated carbon dioxide (CO_2_) concentrations, on the sensitivity of weeds to herbicides. Here, glyphosate response of both *Conyza canadensis* and *Chenopodium album* was evaluated under different environmental conditions. Reduced glyphosate sensitivity was observed in both species in response to increased temperature, elevated CO_2_ level, and the combination of both factors. Increased temperature had greater effect on plant survival than elevated CO_2_ level. In combination, high temperature and elevated CO_2_ level resulted in loss of apical dominance and rapid necrosis in glyphosate-treated plants. To investigate the mechanistic basis of reduced glyphosate sensitivity, translocation was examined using ^14^C-glyphosate. In plants that were subjected to high temperatures and elevated CO_2_ level, glyphosate was more rapidly translocated out of the treated leaf to shoot meristems and roots than in plants grown under control conditions. These results suggest that altered glyphosate translocation and tissue-specific sequestration may be the basis of reduced plant sensitivity. Therefore, overreliance on glyphosate for weed control under changing climatic conditions may result in more weed control failures.

## Introduction

Weeds cause significant crop yield and economic losses in agriculture. Worldwide, the potential loss in overall yield of our major crops due to weeds (34%, on average) is higher than that due to other crop pests, including insects, pathogens, viruses, and animal pests^1,2^. Treatment with herbicides is a highly effective means of controlling weeds as herbicides can kill 90 to >99% of the weeds targeted^3,4^. However, the evolution of herbicide resistance is reducing the overall efficacy of chemical weed management. Presently, more than 250 herbicide-resistant weed species and almost 500 unique cases of resistance have been reported^5^.

Changing environmental conditions are expected to have major effects on plant physiological processes such as stomatal conductance, photosynthetic efficiency and growth rate^6^. Negative impacts of climate change on agricultural productivity has been widely recognized, mainly in the form of potential 6–13% decreases in crop yields^7,8^. Mounting evidence suggests that changing climate conditions may also reduce the sensitivity of weeds to some herbicides^2,9,10^.

Glyphosate is the most commonly used herbicide in the world^11^. It has a unique mode of action inhibiting 5-enolypyruvylshikimate-3-phosphate synthase (EPSPS; E.C. 2.5.1.19), a key enzyme in the biosynthesis of aromatic amino acids. Glyphosate was found to be less effective under either high temperatures [e.g. in *Conyza canadensis*^12^] or elevated carbon dioxide (CO_2_) levels [e.g. in *Chenopodium album*, *Cirsium arvense*^13,14^ and *Glycine max*^15^] but no studies, to our knowledge, have examined the joint effects of both increased temperature and elevated CO_2_ level on plant response to glyphosate. Reduced glyphosate efficacy is mainly correlated with changes in the translocation and distribution of the herbicide. Vacuolar sequestration, limited cellular uptake and rapid necrosis were all found to play a role in reduced plant sensitivity to glyphosate^16^.

Even though photosynthesis is not the primary inhibitory target of glyphosate, it has been reported to be affected by this herbicide. Glyphosate was suggested to cause inhibition of photosynthetic CO_2_ assimilation^17^ as well as a decrease in intermediates of the photosynthetic carbon reduction cycle^18^. Shikimic acid, one of the main products in the EPSPS pathway, is a precursor of pigments, defense compounds, lignin and other important molecules in plants^19^. Interestingly, glyphosate injury was also found to be correlated with chlorophyll content^20,21^.

This research was conducted to examine the joint effects of increased temperature and elevated CO_2_ level on the sensitivity of weeds to glyphosate. To accomplish this objective, we chose two weed species, *C. canadensis* and *C. album*, that differ in leaf surface characteristics, flowering phenology and plant architecture. The specific research objectives were (1) to examine the influence of increased temperatures, elevated CO_2_ levels, and the combination of both factors on the sensitivity of *C. canadensis* and *C. album* to glyphosate and (2) to investigate the mechanistic basis of plant response to glyphosate treatment under these environmental conditions.

## Results

### Plant response to glyphosate

Plant sensitivity to glyphosate was reduced under high temperatures and elevated CO_2_ levels (Table 1). For both species and all populations, plant survival was highest under the combined high temperature and elevated CO_2_ (HT/ECO_2_) treatment. Two out of four *C. canadensis* populations (CC4 and CC8) had a considerably higher percentage of plants surviving treatment with glyphosate under the combination of low temperature and ambient CO_2_ level (LT/ACO_2_) than all others (Table 1). Thus, differences in plant survival between the LT/ACO_2_ and the HT/ECO_2_ were not statistically significant for these two populations. However, for the remaining six populations of both *C. album* and *C. canadensis*, the survival percentage differed significantly between the LT/ACO_2_ and HT/ECO_2_ treatments (Table 1). Large differences in plant survival between current and projected environmental conditions were recorded for populations CA1, CA3 (*C. album*) and CCS (*C. canadensis*) in which no plants survived glyphosate treatment under LT/ACO_2_ but 61.1%, 69.0% and 64.0% of the plants tested, respectively, survived under HT/ECO_2_ conditions (Table 1). In addition, a higher percentage of glyphosate-treated plants survived under high temperature (HT/ACO_2_) than under elevated CO_2_ level (LT/ECO_2_).

**Table 1:** *Chenopodium album* (CA) and *Conyza canadensis* (CC) populations sampled in California and the percentages of plants surviving glyphosate treatment at the labeled field rate under different environmental conditions.

Pop’ ID	County	Habitat	Latitude (N)	Longitude (W)	LT/ACO_2_^a^	LT/ECO_2_^b^	HT/ACO_2_^c^	HT/ECO_2_^d^
					% Survived (n^e^)	% Survived (n)	% Survived (n)	% Survived (n)
CA1	Yolo	Organic field	38.563	121.738	0 (55) [0,10]^f^ c^g^	8.3 (60) c	34.3 (32) b	61.1 (30) [49;73] a
CA3	San Joaquin	Uncultivated field	38.115	121.464	0 (56) [0;9]	n/t^h^	n/t	69.0 (38) [59;79]
CA4	Madera	Deserted field	37.103	120.257	9.4 (44) [0;19]	n/t	n/t	58.4 (44) [49;68]
CA14	Yolo	Organic field	38.541	121.764	3.1 (44) [0,13]	n/t	n/t	76.9 (39) [67;87]
CCS	Fresno	Orchard	36.802	119.667	0 (47) [0;12] c	19.5 (41) bc	40.8 (49) b	64.0 (76) [53;74] a
CC2	Yolo	Organic field (canal)	38.738	122.045	8.7 (80) [4;17]	n/t	n/t	65.6 (64) [53;76]
CC4	Yolo	Roadside	38.708	122.055	22.2 (54) [13;35]	n/t	n/t	46.0 (50) [33;60]
CC8	San Joaquin	Uncultivated field	37.874	121.252	16.1 (62) [9;28]	n/t	n/t	39.0 (64) [28;51]

^a^Low temperature (18/12 °C) combined with ambient CO_2_ (400 ppm).

^b^Low temperature (18/12 °C) combined with elevated CO_2_ (720 ppm).

^c^High temperature (32/26 °C) combined with ambient CO_2_ (400 ppm).

^d^High temperature (32/26 °C) combined with elevated CO_2_ (720 ppm).

^e^Number of treated plants.

^f^95% confidence intervals are shown in brackets.

^g^Different lowercase letters indicate statistically significant differences among different environmental conditions as determined by a Tukey-Kramer HSD test (α = 0.05).

^h^Not tested.

**Figure 1:**
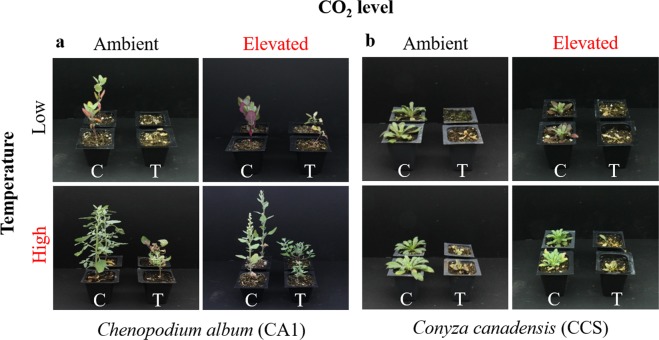
Plant response to glyphosate under different environmental conditions. Glyphosate-treated (T) and untreated (C) plants from population CA1 of *C*. *album* (**a**) and population CCS of *C*. *canadensis* (**b**) grown under different temperatures and CO_2_ levels. Photographs were taken at 21 days after treatment (DAT). Low temperature = 18/12 °C, high temperature = 32/26 °C, ambient CO_2_ = 400 ppm and elevated CO_2_ = 720 ppm.

Loss of apical dominance and outgrowth of multiple lateral shoots were observed in glyphosate-treated plants grown under high temperature (HT/ACO_2_) alone and the combination of both high temperature and elevated CO_2_ level (HT/ECO_2_). This phenotype was consistently observed for *C. album* (Fig. 1a), but only approximately 10% of the *C. canadensis* plants exhibited a loss of apical dominance under HT/ACO_2_ and HT/ECO_2_. Despite using the same photoperiod (11-h) for all treatments, variation in flowering phenology among *C. album* plants under different temperatures was detected. At the end of the experiment, 21 days after glyphosate treatment, both treated and untreated CA1 plants grown under HT/ACO_2_ or HT/ECO_2_ conditions had flower buds or flowers while plants grown under LT/ACO_2_ and LT/ECO_2_ did not have visible reproductive structures (Fig. 1a).

### SPAD measurements

**Figure 2:**
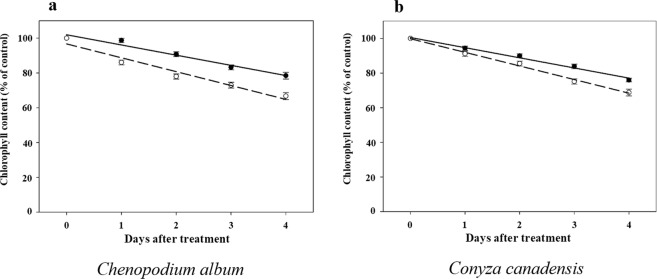
Leaf chlorophyll content (estimated in SPAD units) of glyphosate-treated *C*. *album* (**a**) and *C*. *canadensis* (**b**) plants grown under different environmental conditions over the course of four days after glyphosate application. Solid line - low temperature (18/12 °C) combined with ambient CO_2_ (400 ppm); dashed line - high temperature (32/26 °C) combined with elevated CO_2_ (720 ppm). Error bars represent 95% confidence intervals for the mean response.

Over the four days following herbicide treatment, leaves of glyphosate-treated plants grown under HT/ECO_2_ exhibited more rapid reduction in chlorophyll content (estimated in SPAD units) than leaves of plants grown under LT/ACO_2_ (Fig. 2). The differences in SPAD measurements between the two environmental treatments were statistically significant for both *C. album* and *C. canadensis* (Table 2). Interestingly, differences between species were also observed as glyphosate-treated *C. album* plants grown under HT/ECO_2_ exhibited faster reduction in chlorophyll (Fig. 2a) than *C. canadensis* plants treated and grown under the same conditions (Fig. 2b). Five days after glyphosate application, leaves of treated plants grown under HT/ECO_2_ exhibited severe chlorosis and turgor loss thus preventing further measurements.

**Table 2:** Linear regression of chlorophyll content (estimated in SPAD units) in leaves of glyphosate-treated *Chenopodium album* and *Conyza canadensis* plants as a function of days after treatment with glyphosate.

Species	Treatment^b^	*n*	Parameter estimates^a^		*P*-value
			y-intercept	*b*	
*Chenopodium album*	LT/ACO_2_	324	102.03 [100.08;103.98]	−5.88 [−5.03;−6.73]	<0.001
	HT/ECO_2_	304	96.84 [94.74;98.94]	−8.07 [−7.17;−8.96]	<0.001
*Conyza canadensis*	LT/ACO_2_	296	100.55 [98.90;102.19]	−5.87 [−5.21;−6.53]	<0.001
	HT/ECO_2_	292	99.80 [97.70;101.91]	−7.85 [−6.98;−8.72]	<0.001

^a^95% confidence intervals are shown in brackets.

^b^LT/ACO2 = low temperature (18/12 °C) combined with ambient CO_2_ (400 ppm); HT/ECO2 = high temperature (32/26 °C) combined with elevated CO_2_ (720 ppm).

### Absorption and translocation of ^14^C-glyphosate

**Figure 3:**
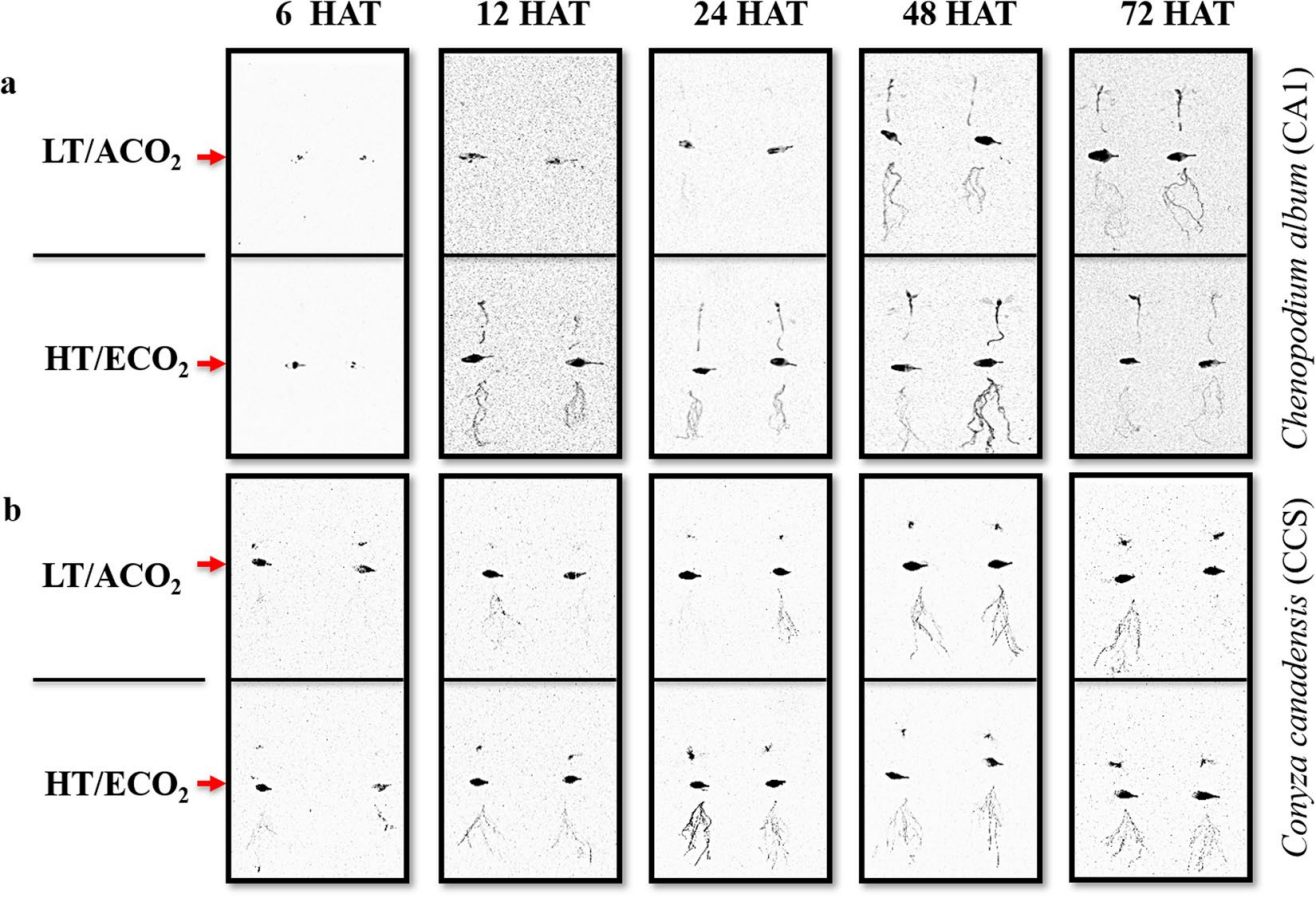
Phosphor images of ^14^C-glyphosate translocation in plants of *C*. *album* (**a**) and *C*. *canadensis* (**b**) grown under different environmental conditions and harvested at 6, 12, 24, 48 and 72 hours after treatment (HAT) with glyphosate. LT/ACO_2_ - low temperature (18/12 °C) combined with ambient CO_2_ (400 ppm), HT/ECO_2_ - high temperature (32/26 °C) combined with elevated CO_2_ (720 ppm). Plants were divided into three parts: treated leaf (indicated horizontally by the red arrow), shoot (above treated leaf), and roots (below treated leaf) prior to imaging.

Phosphor images of ^14^C-glyphosate translocation from the treated leaf to the rest of the plant revealed differences in the distribution of glyphosate within plants grown under different environmental conditions (Fig. 3). For both species, differences in glyphosate translocation were mainly observed at 12, 24 and 48 hours after treatment (HAT). Higher ^14^C-glyphosate signal intensity was detected in the shoot and roots of *C. album* plants grown under HT/ECO_2_ than plants grown under LT/ACO_2_ conditions at both 12 and 24 HAT (Fig. 3a). A similar pattern of glyphosate distribution was observed in *C. canadensis* although the differences in glyphosate distribution among plants grown under the different environmental conditions were not as visually distinguishable as in *C. album* plants (Fig. 3b). For both species, apparent differences in glyphosate translocation were also observed at 48 HAT.

Based on the phosphor imaging results described above, which indicate that the largest differences in ^14^C-glyphosate translocation between plants grown under different environmental conditions, occur at 12, 24 and 48 HAT, we investigated the absorption and quantified the distribution of ^14^C-glyphosate in different plant parts of *C. album* and *C. canadensis* under different environmental conditions (LT/ACO_2_ and HT/ECO_2_) at these time points.

**Figure 4:**
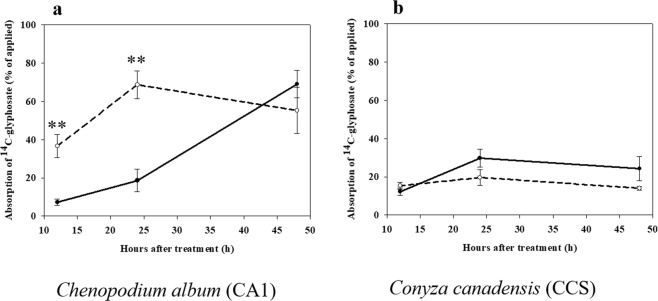
^14^C-glyphosate absorption in plants of *C. album* (**a**) and *C. canadensis* (**b**) grown under different environmental conditions and harvested at 12, 24 and 48 hours after treatment (HAT) with glyphosate. Solid line - low temperature (18/12 °C) combined with ambient CO_2_ (400 ppm), dashed line - high temperature (32/26 °C) combined with elevated CO_2_ (720 ppm). Two asterisks indicate a statistically significant difference (*P* ≤ 0.01) in ^14^C-glyphosate quantity between environmental treatments at different harvest time points. Error bars represent 95% confidence intervals for the mean response.

Glyphosate absorption differed markedly between the two species (Fig. 4). *C*. *album* plants grown under HT/ECO_2_ conditions absorbed ^14^C-glyphosate in a significantly greater amount than plants grown under LT/ACO_2_ within 12 and 24 HAT (Fig. 4a). However, at 48 HAT, no statistically significant difference in glyphosate absorption was observed between plants grown under the different environmental conditions. Although less ^14^C-glyphosate was absorbed by *C*. *canadensis* plants grown under HT/ECO_2_, differences in absorption between plants grown under different environmental conditions (LT/ACO_2_ and HT/ECO_2_) were not statistically significant (Fig. 4b). Overall, *C*. *album* absorbed substantially more ^14^C-glyphosate than *C. canadensis*.

**Figure 5:**
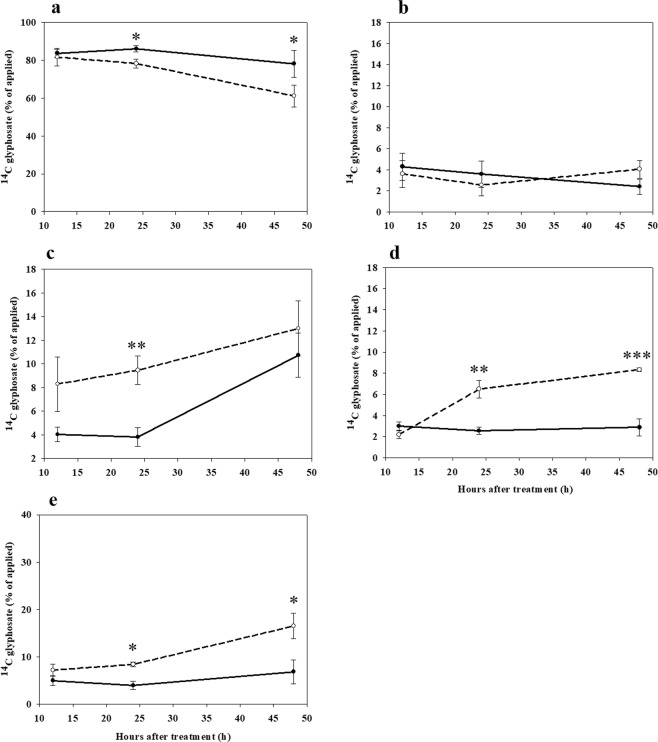
Quantification of ^14^C-glyphosate in the treated leaf (**a**), leaves + petioles (**b**), stem (**c**), shoot apical meristem including young undeveloped leaves (**d**) and roots (**e**), of *C. album* plants. Plants were grown under different environmental conditions and harvested at 12, 24 and 48 hours after treatment (HAT) with glyphosate. Solid line - low temperature (18/12 °C) combined with ambient CO_2_ (400 ppm), dashed line - high temperature (32/26 °C) combined with elevated CO_2_ (720 ppm). One, two, or three asterisks indicate a difference in ^14^C-glyphosate quantity between environmental treatments at different harvest time points, *P* ≤ 0.05, 0.01 and 0.001, respectively. Error bars represent 95% confidence intervals for the mean response.

Quantification of ^14^C-glyphosate translocation into different plant parts revealed that significantly more glyphosate was retained in the treated leaf of *C*. *album* plants grown under LT/ACO_2_ than HT/ECO_2_ conditions at both 24 and 48 HAT (Fig. 5a). In foliage leaves (i.e., all leaves except the treated leaf), low amounts of ^14^C-glyphosate were found in plants grown under both LT/ACO_2_ and HT/ECO_2_ with no statistically significant differences between the environmental conditions (Fig. 5b). Higher amounts of ^14^C-glyphosate were found in plant stems under HT/ECO_2_ than LT/ACO_2_ although a statistically significant difference between treatments was only observed at 24 HAT (Fig. 5c). More glyphosate was found in both the shoot apical meristems (Fig. 5d) and the roots (Fig. 5e) of plants grown under HT/ECO_2_ compared with plants grown under LT/ACO_2_. For both shoot apical meristems and roots, differences between environmental conditions were statistically significant at 24 and 48 HAT (Fig. 5d,e).

**Figure 6:**
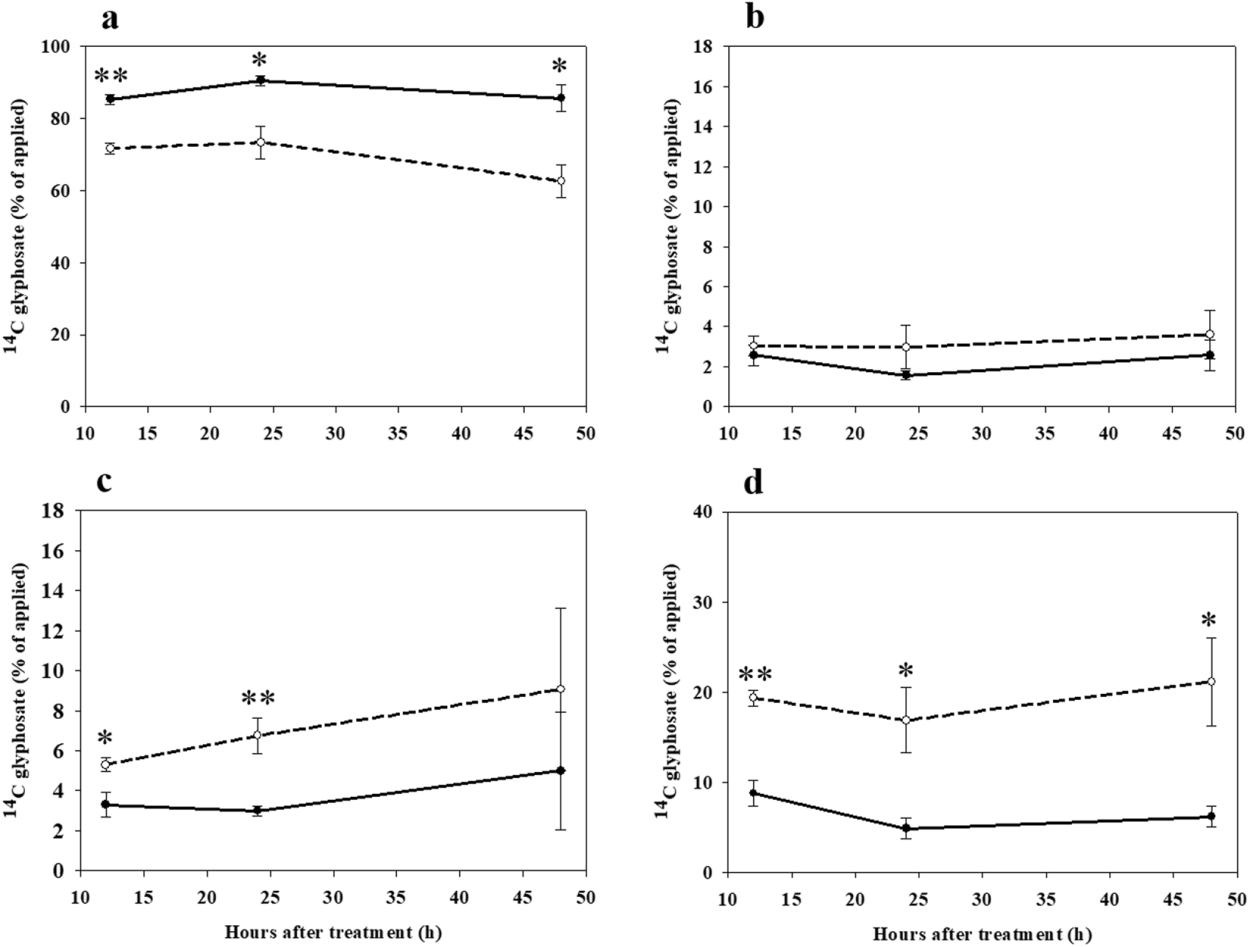
Quantification of ^14^C-glyphosate in the treated leaf (**a**), rosette leaves (**b**), shoot meristems including young undeveloped leaves (**c**) and roots (**d**), of *C. canadensis* plants. Plants were grown under different environmental conditions and harvested at 12, 24 and 48 hours after treatment (HAT) with glyphosate. Solid line - low temperature (18/12 °C) combined with ambient CO_2_ (400 ppm), dashed line - high temperature (32/26 °C) combined with elevated CO_2_ (720 ppm). One or two asterisks indicate a difference in ^14^C-glyphosate quantity between environmental treatments at different harvest time points, *P* ≤ 0.05 and 0.01, respectively. Error bars represent 95% confidence intervals for the mean response.

In *C*. *canadensis*, significantly more ^14^C-glyphosate was translocated out of the treated leaf of plants grown under HT/ECO_2_ at all harvest time points (Fig. 6a). No significant differences were observed in the amount of ^14^C-glyphosate found in the rosette leaves (i.e., all leaves except the treated leaf) of plants grown under different environmental conditions (Fig. 6b). However, more ^14^C-glyphosate was observed in both shoot meristems and the roots of plants grown under HT/ECO_2_ compared with plants grown under LT/ACO_2_ at all harvest time points (Fig. 6c,d). Significant differences in the quantity of ^14^C-glyphosate between environmental conditions were observed at both 12 and 24 HAT for shoot meristems (Fig. 6c), whereas in the roots, significant differences were observed for all harvest time points (Fig. 6d).

## Discussion

Taken together, the results of our study clearly indicate that the control of two major weeds in California agriculture by glyphosate could be reduced under the projected changes in climatic conditions. Compared to current conditions, both *C. canadensis* and *C. album* plants were less sensitive to glyphosate under the higher temperatures, elevated CO_2_ levels and the combination of both environmental conditions, which are predicted for the future. To the best of our knowledge, this research provides the first experimental evidence of the joint effects of both high temperatures and elevated CO_2_ levels on weed sensitivity to glyphosate.

Reduced glyphosate sensitivity under high temperature and CO_2_ conditions was observed for all four populations of each species. Although the populations used for this study were primarily chosen from herbicide-free habitats, two *C. canadensis* populations (CC4 and CC8) exhibited a higher percentage of plants surviving glyphosate treatment at low temperature combined with ambient CO_2_ level (LT/ACO_2_) than all others. The wind-mediated seed dispersal, combined with the evolution and spread of glyphosate resistant *C. canadensis* populations across the Central valley of California^22^, may account for the higher percentage of plants surviving glyphosate under current (LT/ACO_2_) conditions.

The rapid reduction in chlorophyll content (estimated in SPAD units), loss of apical dominance, and early initiation of reproductive structures observed in glyphosate-treated plants grown under high temperature combined with elevated CO_2_ level (HT/ECO_2_) provide insights into the mechanistic basis of the reduced plant sensitivity to glyphosate under climate change scenarios. It is generally claimed that glyphosate controls weedy plants by binding to, and inhibiting the EPSPS enzyme, which is essential for the biosynthesis of branched-chain amino acids^11^. Interestingly, several recent studies, in addition to this study, have revealed changes in phenological and physiological plant traits caused by glyphosate. Outgrowth of lateral shoots^23^, delayed flower development^24^ and reduced stomatal conductance^21^, have been observed in response to glyphosate treatment. Additionally, as a phloem-mobile herbicide, glyphosate exhibits a classic source-to-sink translocation pattern^25^. The influence of glyphosate on photosynthesis-related processes, such as carbon fixation, starch accumulation and general carbohydrate formation, can eventually lead to self-induced limitation of glyphosate translocation^26^. Our findings suggest that most of the glyphosate that was not retained in the treated leaves was translocated into shoot apical meristems and young leaves (strong sinks), which caused rapid leaf decay and thus reduced glyphosate translocation to other plant organs.

Glyphosate absorption differed between the two species. In *C. album*, significantly higher ^14^C-glyphosate absorption was observed in plants grown under HT/ECO_2_ compared to LT/ACO_2_ conditions. In *C. canadensis*, ^14^C-glyphosate absorption was marginally higher, but not significantly, under LT/ACO_2_ conditions. However, despite the differences in glyphosate absorption between species, the translocation and distribution pattern of ^14^C-glyphosate within plants, once absorbed, was similar. In glyphosate-treated plants grown under HT/ECO_2_, glyphosate was translocated more quickly out of the treated leaf to other plant tissues than in plants grown under LT/ACO_2_ conditions. Moreover, in plants grown under HT/ECO_2_, glyphosate translocation from the treated leaf into strong sinks (e.g. shoot meristems and roots) was rapid for both *C. album* and *C. canadensis* (Figs 5d,e and 6c,d, respectively). The rapid movement of glyphosate into shoot apical meristems and roots may reduce the mobility of the herbicide to other parts of the plant thereby reducing the overall sensitivity of plants to glyphosate under higher temperature and CO_2_ (HT/ECO_2_) conditions. It has been hypothesized for many glyphosate-resistant weeds that less glyphosate is translocated from the treated leaf to other plant parts compared to glyphosate-sensitive plants^16^. Interestingly, our results suggest a mechanistic basis for reduced plant sensitivity to glyphosate that differs from the altered glyphosate translocation mechanism hypothesized for many glyphosate-resistant weeds. For both *C. album* and *C. canadensis*, reduced translocation of glyphosate from the treated leaf was proposed as the mechanism for glyphosate tolerance^27,28^. Our results suggest that the mechanism leading to reduced glyphosate sensitivity under high temperatures and elevated CO_2_ levels may differ from that conferring evolved glyphosate resistance in weeds.

The pattern of glyphosate translocation observed in *C*. *canadensis* and *C*. *album* in this study can also explain the loss of apical dominance and the initiation of lateral shoots in glyphosate-treated plants grown under HT/ECO_2_ (Fig. 1a). It is well-known that auxin moves basipetally from the apical shoot in order to suppress lateral bud growth^29^. Glyphosate translocation into the shoot apical meristem may cause severe damage to this tissue and, as a result, constrain auxin production. Low quantities of auxin and glyphosate at the whole plant level may enable the outgrowth of lateral shoots which, in turn, could lead to increased plant survival after glyphosate treatment and the phenotype observed in this study.

In conclusion, we have shown that glyphosate-treated plants grown under increased temperature and elevated CO_2_ level exhibit reduced glyphosate sensitivity. Thus, the continued overreliance on glyphosate for weed control under changing climatic conditions may result in more weed control failures. In addition, from a practical point of view, the loss of apical dominance and early initiation of reproductive structures, as observed in glyphosate-treated plants grown under high temperature in this study, could further exacerbate weed problems by resulting in an unexpected increase in seed production per plant and rapid replenishment of the soil seed bank. Our translocation studies have revealed variation in glyphosate distribution pattern between plants grown under different environmental treatments. Tissue-specific glyphosate sequestration may be the leading cause for sub-lethal glyphosate quantities at the whole plant level reducing the overall efficacy of the herbicide. Further research is required to determine the exact mechanism leading to the reduced plant sensitivity to glyphosate under altered environmental conditions.

## Materials and Methods

### Plant material

Four populations of each species, *C. album* and *C. canadensis*, were sampled for seeds across the Central Valley of California in 2017 (Table 1). Seeds were collected from 30 randomly selected plants in each population and pooled. To increase the probability of collecting seeds from glyphosate-sensitive individuals, populations were primarily chosen from crops grown organically and from areas where herbicides are less likely to be used. In addition to the seeds sampled from those fields, seeds of a previously characterized glyphosate-susceptible *C. canadensis* population^30^ were included for comparison.

### Temperature and CO_2_ treatments

Farmers in the region of the central Valley of California usually treat *C. album* and *C. canadensis* with glyphosate after seeds germinate and seedlings emerge in February or March during which the daily current temperatures averaged 18 °C and current maximum temperatures averaged 27 °C. Based on Intergovernmental Panel on Climate Change^31^ predictions^32^, future projected extreme temperatures are estimated to be 3–5 °C higher than current maximum temperatures. Thus, the two temperature treatments chosen for this study were 18/12 °C (day/night) as the current average and 32/26 °C (day/night) as the projected maximum. The projected maximum temperature was calculated by adding 5 °C to the current maximum (27 °C). A difference of 6 °C between day and night temperatures was chosen in accordance with the current day/night temperature difference and with previous studies of *C. album* (Ziska *et al*.^13^) and *C. canadensis* (Kleinman *et al*.^12^).

CO_2_ treatment levels were ambient (400 ppm) and elevated (720 ppm), which corresponds to future climate projections and within the range of CO_2_ levels projected by the year 2100^31,33,34^. Environmentally controlled growth chambers (Conviron-PGR15), equipped with non-dispersive infrared CO_2_ analyzers (Horiba model APBA-250E) and valves injecting pure CO_2_ to the incoming air stream, were set at either the near normal ambient CO_2_ level (400 ppm) or at the elevated CO_2_ level (720 ppm). Chamber CO_2_ concentrations were logged at 30 second intervals and averaged for each 24 h period, showing that CO_2_ levels averaged 490 ± 40 ppm for the ambient treatment and 720 ± 5 ppm for the elevated CO_2_ treatment.

### Plant response to glyphosate

Seeds from each *C*. *album* and *C*. *canadensis* population sampled were germinated in flats filled with commercial potting media (Professional growing mix, SunGro® Horticulture Canada, Ltd., Vancouver, British Columbia, Canada). Seedlings of *C*. *album* at the two- to four-leaf stage and *C*. *canadensis* at the three- to four-leaf stage were transplanted into 5 by 5 cm plastic pots (one plant per pot) filled with the same potting media and grown in a growth chamber set at 25/15 °C (day/night) temperatures and 11-h photoperiod, representative of the day length for February/March in California, and a light intensity of 600 *µ*mol m^−2^ s^−1^ provided by fluorescent and incandescent bulbs. Seedlings were watered daily.

Three days after transplanting, 20–40 healthy seedlings from each population were moved to two growth chambers that differed in the following temperature and CO_2_ conditions: [1] LT/ACO_2_ – low temperature (18/12 °C) combined with ambient CO_2_ (400 ppm), and [2] HT/ECO_2_ – high temperature (32/26 °C) combined with elevated CO_2_ (720 ppm) but with the same photoperiod and light intensity as described above. Seedlings of *C*. *album* were grown to a height of 6–8 cm, whereas seedlings of *C. canadensis* were grown to the 8–10 rosette leaf stage (5–6 cm in diameter), then treated with glyphosate (Roundup PowerMax^®^, Monsanto, St. Louis, MO, USA) at the labeled field rate of 867 g ae ha^−1^ using an automated spray chamber equipped with a flat-fan 8001E nozzle (TeeJet^®^, Spraying Systems Co., Wheaton, IL, USA). The sprayer was calibrated to deliver 187 L ha^−1^ of glyphosate solution at a pressure of 296 kPa. For each treatment, five unsprayed individual plants were designated as untreated controls. One hour after glyphosate treatment, plants were returned to their respective growth chambers. Plant survival was assessed 21 days after treatment (DAT). The experiment was repeated 2–3 times. Treatment combinations and experimental runs were rotated between the two chambers.

In addition, seedlings of two populations (CA1 for *C*. *album* and population CCS for *C*. *canadensis*) were assessed for plant response to glyphosate under two additional temperature and CO_2_ combinations: [3] LT/ECO_2_ – low temperature (18/12 °C) combined with elevated CO_2_ (720 ppm), and [4] HT/ACO_2_ – high temperature (32/26 °C) combined with ambient CO_2_ (400 ppm). Photoperiod, light intensity, glyphosate application and data collection were the same as described above. Due to a shortage of available growth chambers in which CO_2_ levels could be regulated, only one population of each species could be tested at these environmental conditions.

### Chlorophyll content as an indicator of glyphosate’s effect on photosynthesis

For each population of *C. album* and *C. canadensis*, 16 plants grown under LT/ACO_2_ and 16 plants grown under HT/ECO_2_ conditions [eight plants sprayed with glyphosate and eight unsprayed plants (untreated controls)] were measured for leaf greenness and an estimate of chlorophyll content using a portable chlorophyll meter (SPAD 502®, Minolta, Konica Minolta Sensing, Inc., Osaka, Japan), following the method of Yannicccari *et al*.^35^. Three independent measurements were taken at the middle section of the youngest fully expanded leaf four days following treatment with glyphosate. Chlorophyll content (estimated in SPAD units) was calculated as the average of the three measurements and expressed for glyphosate-treated plants as a percentage of the respective values obtained for untreated control plants.

### Absorption and translocation of ^14^C-glyphosate

Glyphosate absorption and translocation under different temperature and CO_2_ conditions was assessed using a completely randomized experimental design with four replicates. Seeds from *C*. *album* population CA1 and *C*. *canadensis* population CCS were germinated and seedlings grown as described above. Seedlings of *C*. *album* at the two- to four-leaf stage and *C*. *canadensis* at the three- to four-leaf stage were transplanted into 40 ml vials and grown hydroponically with a dilute nutrient solution, as described in Moretti and Hanson^28^, in the growth chambers maintained at LT/ACO_2_ and HT/ECO_2_ conditions.

A solution containing glyphosate at a final concentration that approximated an 867 g ae ha^−1^ spray solution at 187 L ha^−1^ carrier volume was prepared by mixing ^14^C-glyphosate (American Radiolabeled Chemicals, Inc., St. Louis, MO, USA, 3700 kBq/mL^−1^) and a commercial formulation of glyphosate (Roundup PowerMax^®^, Monsanto, St. Louis, MO, USA). When plants reached a height of 6–7 cm for *C*. *album* and the 6–8 rosette leaf stage for *C. canadensis*, a 2 µL volume of the solution (approximately 1.6 kBq of ^14^C-glyphosate) was applied to the middle adaxial surface of the youngest fully expanded leaf of each plant using a blunt-edged syringe (Hamilton, Reno, NV, USA) in the form of two droplets, as described in Nandula and Vencill^36^. Plants were harvested at 6, 12, 24, 48 and 72 hours after treatment (HAT). At each harvest time, the treated leaf was removed and gently rinsed for 30 seconds in vials containing 10 mL of distilled water to remove the unabsorbed ^14^C-glyphosate from the leaf surface. After removing the treated leaf, plants were dissected into shoots and roots.

Phosphor image analysis was used to visualize herbicide translocation. ^14^C-glyphosate treated and dissected plant parts (treated leaves, shoots, and roots) were pressed between two layers of paper and dried at 60 °C for 72 h. After cooling to room temperature, each sample was placed in a 20 × 40 cm exposure cassette (GE Healthcare Bio-Sciences Corp., Piscataway, NJ, USA) and brought into contact with a standard storage phosphor screen (GE Healthcare Bio-Sciences Corp., Piscataway, NJ, USA) for 24 h. Glyphosate translocation was visualized using the Storm 860 PhosphorImager system (Molecular Dynamics, Sunnyvale, CA, USA). Image analysis was conducted using the ImageQuant 5.0 software (Amersham Biotech–Molecular Dynamics, Sunnyvale, CA, USA).

Following phosphor image analysis, ^14^C-glyphosate translocation was quantified at three harvest time points, 12, 24 and 48 HAT, for both species. To measure the amount of non-absorbed glyphosate, rinsate (i.e., the treated leaf wash) was evaporated to dryness and resuspended in 10 ml of scintillation cocktail (Ultima Gold^™^, Perkin Elmer, Walthan, MA). Rinsate radioactivity was quantified using a liquid scintillation spectrometer (LSS) device (LS 6500, Beckman Coulter, Fullerton, CA). The oven-dried plant samples used for phosphor image analysis were also used to assess the distribution of ^14^C-glyphosate. Treated leaves and roots were combusted with no further dissection whereas shoots were divided into several subsections as illustrated in Supplementary Fig. S1. For *C. album*, each shoot was divided into three parts: 1) shoot apical meristems including young undeveloped leaves, 2) leaves + petioles below the treated leaf, and 3) stem. For *C. canadensis*, each shoot was divided into two parts: 1) shoot meristems including young undeveloped leaves and 2) the remaining rosette leaves. Different plant parts were placed separately into a combustion cone and dried at 60 °C for 96 h. Each cone was combusted in a biological oxidizer (Sample Oxidizer Model 307, PerkinElmer, Waltham, MA, USA). The evolved ^14^CO_2_ was trapped in 10 ml of a carbon dioxide adsorbent solvent (Carbo-Sorb® E, PerkinElmer, Waltham, MA, USA) and mixed with 10 ml of scintillation cocktail (Permaflour® E + , PerkinElmer, Waltham, MA, USA). Radioactivity was quantified using the LSS device described above.

### Statistical analysis

Data on the survival of glyphosate-treated plants grown under LT/ACO_2_ and HT/ECO_2_ were analyzed using a generalized linear model (GLM) with PROC GENMOD of SAS (ver 9.4., SAS Institute Inc., Cary, NC, USA). The loglikelihood ratio test was used to assess the significance of the interaction between experimental runs and treatments as well as the main effects of experimental run. Probabilities of plant survival and the 95% confidence intervals for all possible combinations of populations by treatment were estimated using the LSMEANS statement of SAS. For populations CA1 and CCS, an additional analysis of plant survival data was conducted across all four temperature and CO_2_ combinations (LT/ACO_2_, LT/ECO_2_, HT/ACO_2_ and HT/ECO_2_). Data were analyzed using ANOVA in JMP (*ver*. 13) statistical package (SAS Institute Inc., Cary, NC, USA). Means were compared using Tukey-Kramer honestly significant difference (HSD) test (α = 0.05).

SPAD measurements were pooled for each species and means were compared in agreement with a Levene’s ANOVA test for homoscedasticity of variance (*P* ≥ 0.05). No outliers were identified with the studentized residuals technique based on a t-distribution with α = 0.05. Normality of residues (Shapiro-Wilk’s test) and homoscedasticity of variance (Fligner-Killeen’s test) were tested with α = 0.05. Multiple linear regressions of leaf greenness as a function of days after treatment with glyphosate were performed separately for each treatment (LT/ACO_2_ and HT/ECO_2_) and weed species, and regression slopes were obtained with their 95% confidence intervals.

For the absorption and translocation studies, total glyphosate quantity was converted into percentages according to equations 1 and 2. *R* signifies the recovered radioactivity.

1 $$recovery\,( \% )=\frac{{R}_{rinsate}+{R}_{allplantsections}}{{R}_{applied}}\times 100$$

2 $$absorption\,( \% \,of\,applied)=\frac{{R}_{applied}-{R}_{rinsate}}{{R}_{applied}}\times 100$$

Translocation of ^14^C-glyphosate to different plant sections was calculated using equation 3 where *R* signifies the recovered radioactivity; *ME*, the shoot meristems including young undeveloped leaves; *LS*, the remaining rosette leaves, *SM*, the stem; *RS*, the roots, and *TL*, the treated leaf.

3 $$percentage\,in\,ME/LS/SM/RS/TL\,=\frac{{R}_{ME/LS/SM/RS/TL}}{{R}_{applied}-{R}_{rinsate}}\times 100$$

Data from absorption and translocation studies were analyzed using ANOVA in JMP (*ver*. 13) statistical package (SAS Institute Inc., Cary, NC, USA). Means were compared using Student’s *t*-test (α = 0.05). Data were visualized separately for each treatment (LT/ACO_2_ and HT/ECO_2_) and weed species using SigmaPlot (*ver*. 12) software (Systat Software Inc., San Jose, CA, USA).

## Supplementary information

Supplementary Information
