# Supplementary material for: Increased temperatures and elevated CO2 levels reduce the sensitivity of Conyza canadensis and Chenopodium album to glyphosate
Source: Sci Rep. 2019 Feb 18;9:2228. doi: 10.1038/s41598-019-38729-x (PMC6379362; doi:10.1038/s41598-019-38729-x)
Supplement: Supplementary file 1 — Supplementary Information [file 41598_2019_38729_MOESM1_ESM.docx]

**Increased temperatures and elevated CO_2_ levels reduce the sensitivity of *Conyza canadensis* and *Chenopodium album* to glyphosate**

Maor Matzrafi^*,1^, Caio Brunharo^1^, Parsa Tehranchian^1,2^, Bradley D. Hanson^1^, Marie Jasieniuk^1^

^1^Department of Plant Sciences, University of California at Davis, Davis, CA 95616, USA

^2^SynTech Research, P.O. Box 700, Sanger, CA 93657, USA

**Correspondance:**

^*^Maor Matzrafi, Department of Plant Sciences, University of California-Davis, Davis, CA 95616, USA.

Email: [mmatzrafi@ucdavis.edu](mailto:mmatzrafi@ucdavis.edu)


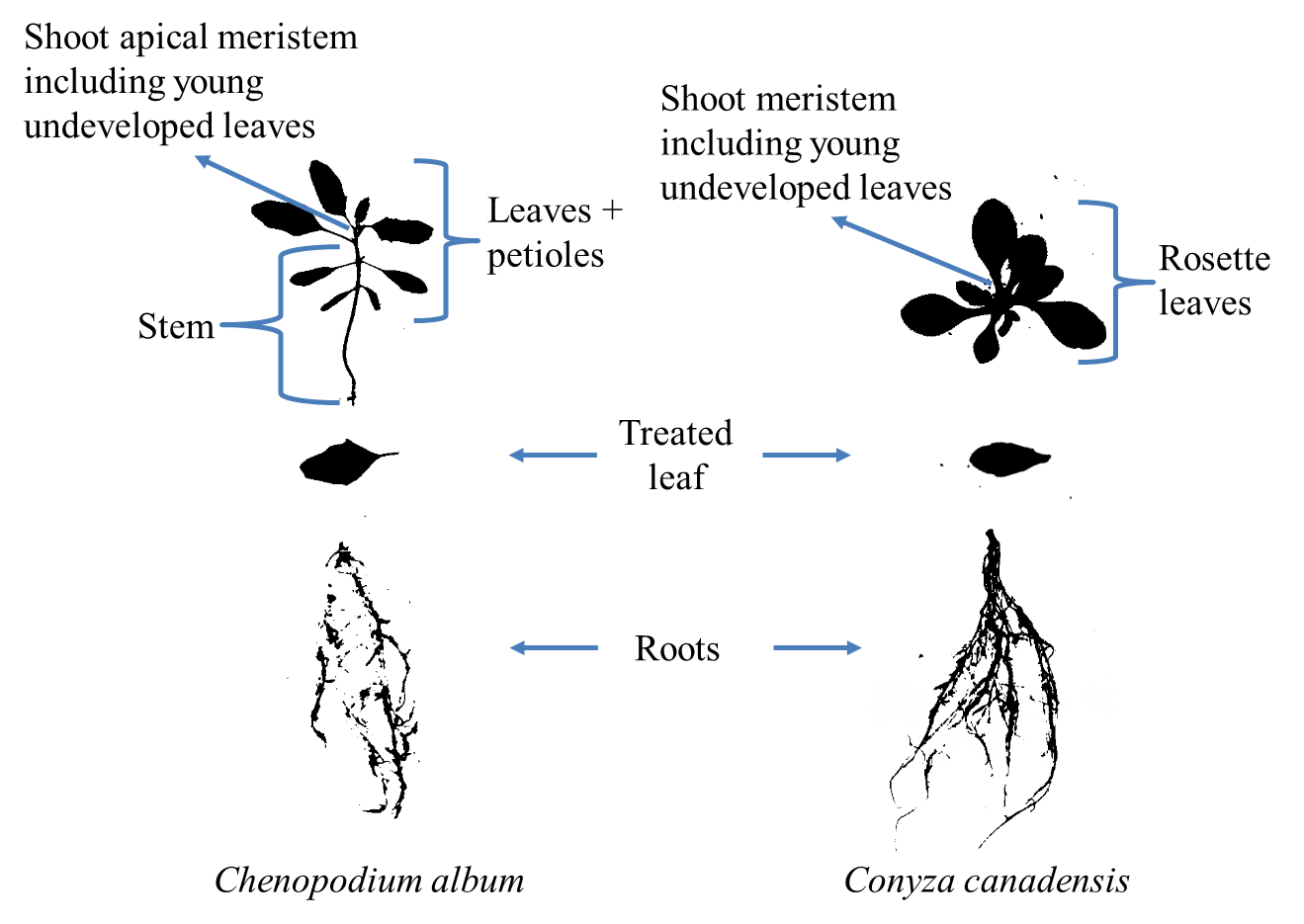


**Supplementary Figure 1.** Illustration of the dissection of *C. album* and *C. canadensis* plants for the ^14^C-glyphosate quantification experiments.
